# Editorial: “Nuclear medicine in cancer diagnosis”

**DOI:** 10.3389/fmed.2022.1035994

**Published:** 2022-09-21

**Authors:** Francesco Cicone, Philipp Lohmann

**Affiliations:** ^1^Nuclear Medicine Unit, University Hospital “Mater Domini”, Catanzaro, Italy; ^2^Department of Experimental and Clinical Medicine, “Magna Graecia” University of Catanzaro, Catanzaro, Italy; ^3^Neuroscience Research Centre, PET/MR Unit, “Magna Graecia” University of Catanzaro, Catanzaro, Italy; ^4^Institute of Neuroscience and Medicine (INM-4), Research Center Juelich (FZJ), Juelich, Germany; ^5^Department of Stereotaxy and Functional Neurosurgery, Faculty of Medicine and University Hospital Cologne, Cologne, Germany

**Keywords:** diagnostic radiopharmaceuticals, nuclear medicine, radionuclide imaging, molecular imaging, tumor detection, PET

In recent years, nuclear medicine had a tremendous impact on the management of patients with cancer, particularly thanks to the widespread use of positron emission tomography (PET) with [^18^F]-fluorodeoxyglucose (FDG). Furthermore, additional fluorinated and non-fluorinated radiopharmaceuticals emerged and have now been integrated in current diagnostic guidelines of several tumors. The landscape of diagnostic radiopharmaceuticals is theoretically limitless, given the infinite number of molecules currently under development or that could be radiolabeled to probe any cellular metabolic and functional aspects of tumors and tumor microenvironment. Owing to the rapidly expanding role of nuclear medicine in cancer diagnosis, a focused Research Topic was launched by the section of Nuclear Medicine of *Frontiers in Medicine*. Between May 2021 and July 2022, ten articles were eventually published within the Research Topic, covering clinical and pre-clinical applications of either well-established or novel radiopharmaceuticals for cancer diagnostics.

Four published articles were dedicated to FDG, the most widely used tracer in oncology. Wang et al. investigated the use of FDG findings to drive ultrasound-guided core biopsy of supraclavicular lymph nodes (SLN) in 54 patients with suspected lung cancer. Diagnosis through SLN has a high potential clinical impact despite minimal invasiveness, as it can directly upstage patients with lung cancer indicating an inoperable disease. The biopsy procedure was performed within 2 days after FGD PET/CT and revealed a diagnostic yield of 98.1%, sparing further bronchoscopy, CT-guided lung biopsy or surgery in 38/54 (70%) patients. In the study conducted by Tien Cong et al. in 86 patients with stage IV lung adenocarcinoma, FDG uptake was found to be higher in patients with positive programmed death-ligand 1 (PD-L1) expression than in patients with PD-L1-negative tumors. This study compares with other previous publications, suggesting that FDG PET could help predict response to PD-1/PD-L1 inhibitors.

One of the most frequent applications of FDG is the investigation of incidental pulmonary nodules. Lai et al. evaluated the potential of a novel deep learning method for an improved differentiation between malignant and benign pulmonary nodules using FDG PET/CT in a group of 112 patients. The authors demonstrated that a deep learning model using high-resolution representation learning might aid in the classification of pulmonary nodules without the need for manual expert annotations. Huang et al. reported on the diagnostic value of FDG PET in the differential diagnosis of pancreatic lesions as compared with contrast-enhanced CT (CE-CT), contrast-enhanced magnetic resonance imaging (CE-MRI) and serum carbohydrate antigen 19-9 (CA 19-9) in a large retrospective cohort of 467 patients. The study showed that FDG PET/CT has higher specificity than CE-CT and CE-MRI, resulting in a higher overall diagnostic accuracy. In addition, FDG was more sensitive and specific than CA 19-9. The authors conclude that the combination of FDG PET/CT with other diagnostic modalities could enhance the diagnostic efficiency, although medium or well-differentiated pancreatic cancers may show low FDG uptake.

Six published articles were dedicated to non-FDG radiopharmaceuticals. Two of these papers focused on the optimization of imaging acquisition and reconstruction of widely available radiopharmaceuticals, such as 3,4-dihydroxy-6-[^18^F]-fluoro-l-phenylalanine (FDOPA) and [^68^Ga]-DOTA-TATE, respectively. The study by Girard et al. assessed the value of dynamic FDOPA PET/CT in 14 patients with newly diagnosed glioma and found an additive value of kinetic parameters over conventional static semiquantitative image assessment. Although the results of the study are interesting, the clinical value of kinetic FDOPA uptake parameters in patients with primary brain warrants confirmation in larger cohorts.

Improvements in PET image reconstruction methods that allow for shorter acquisition times or for a reduction of the injected tracer activity while preserving image quality and accurate quantification are of high clinical relevance. This was accomplished by the total variation regularized expectation maximization (TVREM) image reconstruction method evaluated by Liu et al. in 17 patients with neuroendocrine tumors undergoing [^68^Ga]-DOTA-TATE PET/CT. TVREM could reduce the image noise and increase the signal-to-noise ratio and, hence, seems to be a promising reconstruction algorithm that should be further evaluated for other applications.

Two additional papers reported on novel radiopharmaceuticals, currently at different stages of development. The ability of [^18^F]AlF-NOTA-HER2 to monitor changes of human epidermal growth factor receptor 2 (HER2), an important predictive biomarker in gastric cancer, was demonstrated by Han et al. in a xenograft mouse model. The radiopharmaceutical could be produced with a high radiochemical yield and demonstrated a good image contrast and resolution. Consequently, [^18^F]AlF-NOTA-HER2 may be a promising PET probe for the non-invasive whole-body detection of the HER2 status in patients with gastric cancer.

Prostate-specific membrane antigen (PSMA) represents an attractive target for theragnostic applications in patients with prostate cancer (PC). Due to its favorable characteristics, the PSMA-targeting analog PSMA-617 has been mostly used for therapeutic applications following radiolabelling with ^177^Lu or alpha emitters. In their paper, Qin et al. made diagnostic use of [^68^Ga]-PSMA-617 in 53 patients with suspected PC, assessing the utility of a delayed PET/MR pelvic acquisition about 3 h following radiopharmaceutical injection, in addition to whole-body PET/CT or PET/MRI. The authors concluded that a delayed acquisition is not necessary in most patients with advanced PC, although it may be interesting to further investigate its role in patients with early-stage disease.

Lastly, the Research Topic features two review papers. Ghidaglia et al. conducted a systematic review of dual-tracer FDG/[^18^F]-Choline PET to investigate the level of evidence for a link between tracer uptake and the degree of differentiation of resected hepatocellular carcinoma (HCC). The authors found overlapping uptake behavior between well- and less differentiated HCC, which makes the differentiation based on PET alone challenging. Further studies to improve the usefulness of PET in the clinical management of patients with HCC were recommended.

The inflammatory marker cyclooxygenase-2 (COX-2) was shown to play a role in colon cancer development since the early stages of tumorigenesis. Dagallier et al. reviewed the results of COX-2-targeting radiopharmaceuticals in preclinical models of colon cancer. Despite promising results *in vitro*, none of the radiopharmaceuticals developed so far demonstrated sufficient targeting efficiency *in vivo* to advance to the clinical stage.

In summary, the articles published in this Research Topic present original works, as well as review papers on a diverse range of nuclear medicine diagnostic applications and developments. We sincerely hope that the readers will enjoy and find useful information for their clinical and research activity.

## Author contributions

All authors listed have made a substantial, direct, and intellectual contribution to the work and approved it for publication.

## Conflict of interest

The authors declare that the research was conducted in the absence of any commercial or financial relationships that could be construed as a potential conflict of interest.

## Publisher's note

All claims expressed in this article are solely those of the authors and do not necessarily represent those of their affiliated organizations, or those of the publisher, the editors and the reviewers. Any product that may be evaluated in this article, or claim that may be made by its manufacturer, is not guaranteed or endorsed by the publisher.

